# Effects of Sinomenine on the Expression of microRNA-155 in 2,4,6-Trinitrobenzenesulfonic Acid-Induced Colitis in Mice

**DOI:** 10.1371/journal.pone.0073757

**Published:** 2013-09-16

**Authors:** Qiao Yu, Siying Zhu, Rui Zhou, Fengming Yi, Yuntao Bing, Sha Huang, Zixi Wang, Chunyu Wang, Bing Xia

**Affiliations:** 1 Department of Gastroenterology/Hepatology, Zhongnan Hospital of Wuhan University School of Medicine, Wuhan, P.R. of China; 2 The Hubei Clinical Center and Key Laboratory of Intestinal and Colorectal Diseases, Wuhan, P.R. of China; H.Lee Moffitt Cancer Center & Research Institute, United States of America

## Abstract

**Background:**

Sinomenine, a pure alkaloid isolated in Chinese medicine from the root of *Sinomenium acutum*, has been demonstrated to have anti-inflammatory and immunosuppressive effects. MicroRNAs (miRNAs) are gradually being recognized as critical mediators of disease pathogenesis via coordinated regulation of molecular effector pathways.

**Methodology/Findings:**

After colitis was induced in mice by instillation of 5% (w/v) 2,4,6-trinitrobenzenesulfonic acid (TNBS), sinomenine at a dose of 100 or 200 mg/kg was orally administered once daily for 7 days. We evaluated body weight, survival rate, diarrhea score, histological score and myeloperoxidase (MPO) activity. The mRNA and protein expression levels of miR-155, c-Maf, TNF-α and IFN-γ were determined by quantitative RT-PCR and immunohistochemistry, respectively. Sinomenine (100 or 200 mg/kg)-treated mice with TNBS-induced colitis were significantly improved in terms of body weight, survival rate, diarrhea score, histological score and MPO activity compared with untreated mice. Both dosages of sinomenine significantly decreased the mRNA and protein expression levels of c-Maf, TNF-α and IFN-γ, which elevated in TNBS-induced colitis. Furthermore, sinomenine at a dose of 200 mg/kg significantly decreased the level of miR-155 expression by 71% (*p* = 0.025) compared with untreated TNBS-induced colitis in mice.

**Conclusions/Significance:**

Our study evaluated the effects and potential mechanisms of sinomenine in the anti-inflammatory response via miRNA-155 in mice with TNBS-induced colitis. Our findings suggest that sinomenine has anti-inflammatory effects on TNBS-induced colitis by down-regulating the levels of miR-155 and several related inflammatory cytokines.

## Introduction

Inflammatory bowel disease (IBD) refers to chronic inflammatory disorders of the gut with unknown causes, including Crohn’s disease (CD) and ulcerative colitis (UC) [1], [2]. Although its etiology remains unknown, there is growing evidence that IBD is characterized by dysfunction of the mucosal immunity, a susceptible genetic background and intestinal bacteria that lead to damage of the intestines and colonic mucosa [3]. Some studies have demonstrated that CD is mainly mediated by T helper type 1 (Th1) cells, which secrete interferon-γ (IFN-γ) and tumor necrosis factor (TNF) [4]–[6]. Animal models of colitis have confirmed this viewpoint, including a model of hapten-induced colitis in which 2,4,6-trinitrobenzenesulfonic acid (TNBS) is delivered intrarectally to rodents. This model demonstrates the Th1 activity of local CD4^+^ T cells and is thought to closely resemble CD [7], [8].

MicroRNAs (miRNAs) are a group of small (18–24 nucleotides), endogenous, non-coding single-stranded RNAs that regulate gene expression by controlling the stability and translation of protein-coding mRNAs [9]–[11]. MiR-155 is encoded within a region known as bic, the B-cell integration cluster located on chromosome 21 [12]. The increased expression of miR-155 has been reported in many inflammatory diseases, including UC and CD [13], [14]. Activated mature B and T lymphocytes express miR-155, while studies using miR-155-knockout mice have directly linked miR-155 to the functions of the immune system [15], [16].

Sinomenine (7,8-didehydro-4-hydroxy-3,7-dimethoxy-17-methylmorphinan-6-one) is a pure alkaloid extracted from the stem of the Chinese herbal plant, *Sinomenium acutum* (Rehder and Wilson) (Family Menispermaceae), which has traditionally been used in China and Japan to treat various rheumatic and arthritic diseases [17], [18]. Many previous studies have demonstrated that sinomenine has anti-inflammatory and immunosuppressive effects [19]–[21]. Cheng et al [22] demonstrated that sinomenine attenuates TNBS-induced colitis and that the therapeutic mechanism might be related to the reduction of up-regulated colonic TNF-α and IFN-γ. In the present study, we further evaluated the influence of sinomenine on miR-155, c-Maf, TNF-α and IFN-γ expression levels and investigated the possible mechanisms of sinomenine involving miR-155 in TNBS-induced colitis in mice.

## Materials and Methods

### Ethics Statement

This study was performed in strict accordance with the recommendations in the Guide for the Care and Use of Laboratory Animals of Wuhan University. The protocol was approved by the Committee on the Ethics of Animal Experiments of Wuhan University (Permit Number 2011032). All of the surgeries were performed under sodium pentobarbital anesthesia, and all efforts were made to minimize suffering.

### Animals

Male BALB/c mice were purchased from the Experimental Animal Center of Wuhan University. The animals were 6–8 weeks of age, weighed 18–22 g, and were kept in a specific pathogen-free environment. They were maintained in plastic cages with free access to pellet food and water at 21±2°C and kept on a 12-h light/dark cycle. They were allowed to acclimate to these conditions for at least 5 days before the start of the experiment.

### Induction of Colitis and Treatment

Colitis was induced by a single intracolonic injection into the distal colon of 0.5 mg of the hapten reagent TNBS (Sigma, USA) dissolved in 50% ethanol/phosphate-buffered saline (PBS) solution. The TNBS concentration was determined in previous dose finding studies for BALB/c mice [23]. The mice were anesthetized slightly with an intraperitoneal injection of pentobarbital sodium (40 mg/kg); then, a rubber catheter lubricated with cosmoline was inserted into the colon through the anus. An enema of 100 µL of TNBS was injected only once when the tip of the catheter was 4 cm inside the anus. The mice were held in a vertical position for 1 min after the intrarectal injection. The vehicle group received 100 µL of 50% ethanol-PBS by rectal administration.

The various doses of sinomenine (Zhengqing Pharmaceuticals, Hunan, China) were dissolved in 100 µL of physiological saline. Sinomenine (100 mg/kg and 200 mg/kg) and physiological saline were freshly prepared and administered orally (by gavage) in a volume of 100 µL at 2 h following injection and daily thereafter (Table 1). The mice were observed beginning on day 1 for 7 days. Body weights, diarrhea scores and survival rates were monitored before the induction of colitis and daily thereafter. At the end of the experiment, the mice were sacrificed by cervical dislocation under anesthesia.

**Table 1:** The detailed experimental design in the study.

Group	Group size (n)	Induction and Route	Treatment and Route
Vehicle	15	50% ethanol-PBS, IP	Physiological saline, PO
TNBS	15	TNBS, IP	Physiological saline, PO
100 mg/kg sinomenine	15	TNBS, IP	100 mg/kg sinomenine, PO
200 mg/kg sinomenine	15	TNBS, IP	200 mg/kg sinomenine, PO

Animals were divided into four experimental groups of vehicle, TNBS, 100 mg/kg sinomenine, and 200 mg/kg sinomenine group. PO oral, IP intraperitoneal.

### Assessment and Histological Score of TNBS-Induced Colitis

Diarrhea severity was scored as follows: 0, normal; 2, loose stools; and 4, diarrhea. After 6 days, the mice were sacrificed, and their entire colons were removed from the cecum to the anus and then flushed with phosphate buffer. Colon specimens located 2 cm above the anal verge were achieved. One section of the specimen was fixed overnight in 4% paraformaldehyde and embedded in paraffin, and then sections stained with hematoxylin and eosin were examined. We assigned the histological score according to the following criteria: 0, no signs of inflammation; 1, low levels of leukocyte infiltration; 2, moderate levels of leukocyte infiltration; 3, high levels of leukocyte infiltration, high vascular density and thickening of bowel wall; and 4, transmural infiltrations, loss of goblet cells, high vascular densities and thickening of bowel wall. The other sections of the colons were stored at −80°C for mRNA, protein, and other analyses.

### Measurement of MPO Activity

The myeloperoxidase (MPO) activity assay was performed within one week of the collection of the colonic tissues according to the instructions of the MPO assay kit (Jiancheng BioEngineering, Nanjing, China). The absorbance was measured at 460 nm using a Life Science UV/Vis Spectrophotometer DU 530 (Beckman Coulter, USA). The MPO activity was expressed in units per gram of tissue, and 1 U corresponded to the activity required to degrade 1 mmol of hydrogen peroxide per minute at 25°C.

### Quantitative RT-PCR Analysis

For c-Maf, TNF-α and IFN-γ quantification, total RNA was extracted from colon samples using the TRIzol reagent (Invitrogen, USA) according to the manufacturer’s protocol. The concentrations of total RNA were determined using a spectrophotometer (Nanodrop 2000, Thermo Scientific, USA). cDNA was synthesized using a cDNA Synthesis Kit (GeneCopoeia, USA) for first-strand synthesis. β-actin served as the endogenous control. For miR-155 detection, total RNA was also isolated from colonic tissues using the TRIzol reagent. Quantitative RT-PCR analysis for miR-155 expression (Primer ID, Mumq-0189) was performed using the All-in-One™ miRNA quantitative RT-PCR Detection Kit (GeneCopoeia, USA) [24], [25]. For this assay, 2 µg of total RNA was reverse transcribed to cDNA using a polyA tailing assay. U6 small nuclear RNA (U6, Primer ID, hsnRNA U6) served as the endogenous control. The T_m_ (°C) of the quantitative RT-PCR for the genes was 58°C, and the number of cycles was 30. The gene-specific primers are listed in Table 2. All of the quantitative RT-PCR reactions with SYBR Green (GeneCopoeia, USA) were performed on a SLAN real-time PCR Detection System (GeneCopoeia, USA). The cycle threshold (Ct) indicated the fractional cycle number at which the PCR product was first detected above a fixed threshold. The 2^−ΔΔCT^ method was used to calculate the expression levels of the reported gene mRNAs and miRNAs relative to their respective endogenous controls.

**Table 2:** The gene-specific primer list in the study.

Primer names	Primer sequence (5′–3′)
c-Maf-F	AGAGGCGGACCCTGAAAAA
c-Maf-R	GTGTCTCTGCTGCACCCTCTT
TNF-α-F	AGCACAGAAAGCATGATCCG
TNF-α-R	CTGATGAGAGGGAGGCCATT
IFN-γ-F	TCAAGTGGCATAGATGTGGAAGAA
IFN-γ-R	TGGCTCTGCAGGATTTTCATG
β-actin-F	CTAGGCACCAGGGTGTGAT
β-actin-R	TGCCAGATCTTCTCCATGTC

### Immunohistochemistry

Protocols for immunohistochemistry were described previously [26]. Paraffin-embedded mouse colons were sectioned at a thickness of 4 µm. The tissue sections were deparaffinized in xylene and rehydrated with graded ethanol, and the endogenous peroxidase was inactivated using 0.3% hydrogen peroxide for 10 min. All of the following procedures were performed using two-step anti-rabbit/mouse Histostain™-Plus Kits (K5007, DAKO, Denmark) according to the manufacturer’s instructions. Briefly, the sections were boiled in EDTA (1 mmol/L, pH 6.0) for 10 min in a microwave oven for antigen retrieval. After rinsing with phosphate buffered saline (PBS), the sections were first incubated with goat normal non-immune serum for 15 min. Subsequently, the slides were incubated with a mouse anti-c-Maf antibody (Biosynthesis Biotechnology, Beijing, China) diluted in PBS (1∶100) at 37°C for 2 h. The slides were sequentially incubated with a biotinylated secondary antibody at 37°C for 30 min, followed by incubation with a streptavidin-horseradish peroxidase tertiary antibody at 37°C for 30 min. Finally, the color was developed using 3,3**′**-diaminobenzidine (DAB), and all of the slides were counterstained in hematoxylin before dehydrating and mounting the sections. For negative controls, the sections were incubated in PBS without the anti-c-Maf antibody under the same experimental conditions. For TNF-α and IFN-γ detection, the slides were incubated with a mouse anti-TNF-α antibody (1∶100, eBioscience, USA) and a mouse anti-IFN-γ antibody (1∶100, BioLegend, USA) as previously stated. The expression of c-Maf was localized to intestinal villi epithelial cells and portions of connective tissue cells, while TNF and IFN were mainly expressed in intestinal villi connective tissue. Five 400× images from each group within the intestinal villi area were selected for taking photos. The cumulative values of the integrated optical density (IOD) were analyzed using the Image-Pro Plus 6.0 software. The average value of the IOD was expressed as the mean ± standard deviation (SD).

### Statistical Analysis

The results were expressed as the mean ± SD. The statistical analysis was conducted using SPSS 17.0 software (SPSS for Windows version 17.0, USA). A comparison between the two groups was made using a one-way analysis of variance (ANOVA). The correlations of miR-155 expression with the c-Maf, TNF-α and IFN-γ expression levels were tested using Pearson’s correlation coefficient. Differences were considered significant at *p*<0.05.

## Results

### Effects of Sinomenine on Body Weights, Survival Rates and Diarrhea Scores in Mice with TNBS-induced Colitis

**Figure 1:**
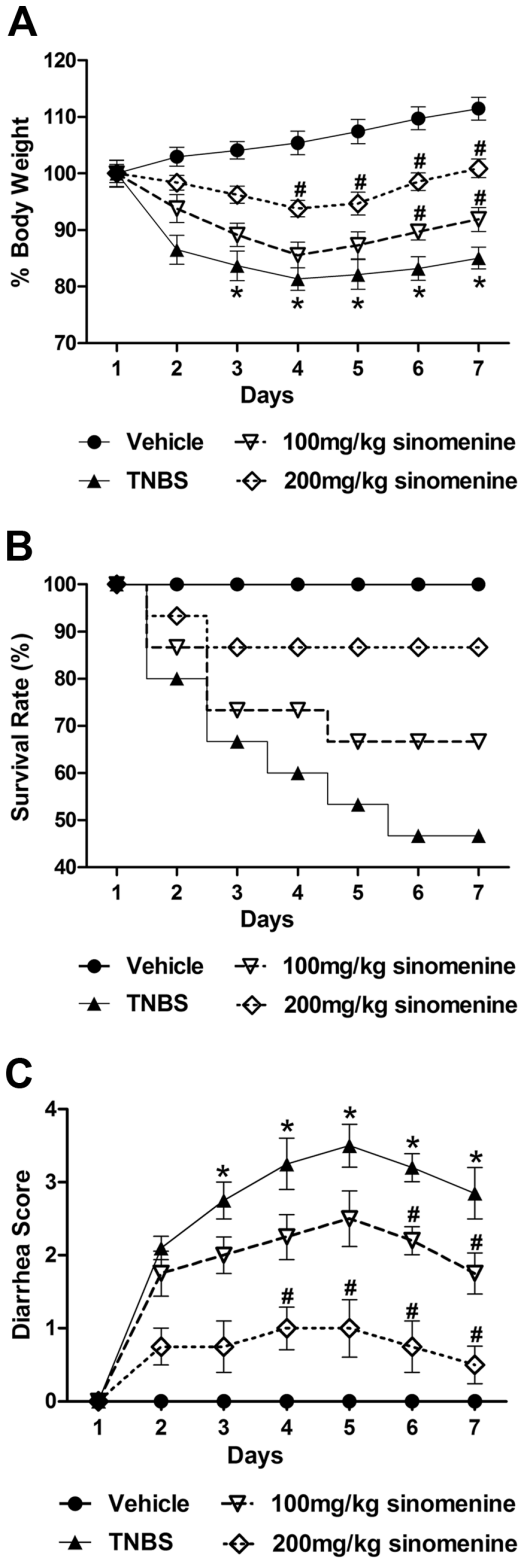
Effects of sinomenine on body weight, survival rate and diarrhea score in TNBS-induced colitis in mice. A. Efficacy of sinomenine on body weight. Body weight increased during administration of different doses (100 and 200 mg/kg) of sinomenine over 7 days. **B. Improvement of survival rate in TNBS-induced colitis by sinomenine.** TNBS-induced colitis resulted in animal death over the course of the whole experiment when compared with the vehicle group, however the sinomenine group showed a much lower mortality rate. **C. Effect on diarrhea signs of colonic inflammation by sinomenine.** TNBS-induced colitis showed serious diarrhea symptoms until day 6, but administration of sinomenine significantly reduced the diarrhea score over the course of the experiment. n = 15 per group, *p<0.05 versus the vehicle group and ^#^ *p*<0.05 versus the TNBS group.

Administration of TNBS resulted in a severe illness characterized by weight loss and decreased survival accompanied by an elevated diarrhea score. As shown in Figure 1, the disease reached a peak on days 4–5 compared with the vehicle group (*p*<0.05).

The difference in body weight for each mouse was determined daily by comparing the current weight with the weight on Day 1, and the animal survival rate was also observed. The body weights and survival rate for the TNBS group were significantly decreased compared with the vehicle group; however, the mice that were treated with sinomenine experienced significant recoveries with respect to body weight and survival rate when compared to the TNBS group. The body weights of the mice treated with 200 mg/kg sinomenine had significantly increased by day 4, and for the mice treated with 100 mg/kg sinomenine, their body weights had significantly increased by day 6 (Figure 1A). As shown in Figure 1B, at the end of the experiment, the numbers of dead mice were 0 (0/15), 8 (8/15), 5 (5/15) and 2 (2/15) for the vehicle group, TNBS group, 100 mg/kg sinomenine group and 200 mg/kg sinomenine group, respectively. It was clear that the body weight loss and mortality rates in the 200 mg/kg sinomenine group were less than those in the TNBS group.

The stool consistency of the mice was monitored every day during the experiment. As shown in Figure 1C, the TNBS group had significantly elevated diarrhea scores compared with the vehicle group, but sinomenine treatment reduced the diarrhea scores in a dose-dependent manner (*p*<0.05). The diarrhea scores in the 200 mg/kg sinomenine group decreased significantly by day 4, but the decrease did not take effect until day 6 in the 100 mg/kg sinomenine group (Figure 1C). Furthermore, sinomenine was observed to relieve the clinical symptoms of the TNBS-induced colitis in a dose-dependent manner.

### Effects of Sinomenine on Histological Analysis and MPO Activity Measurements

**Figure 2:**
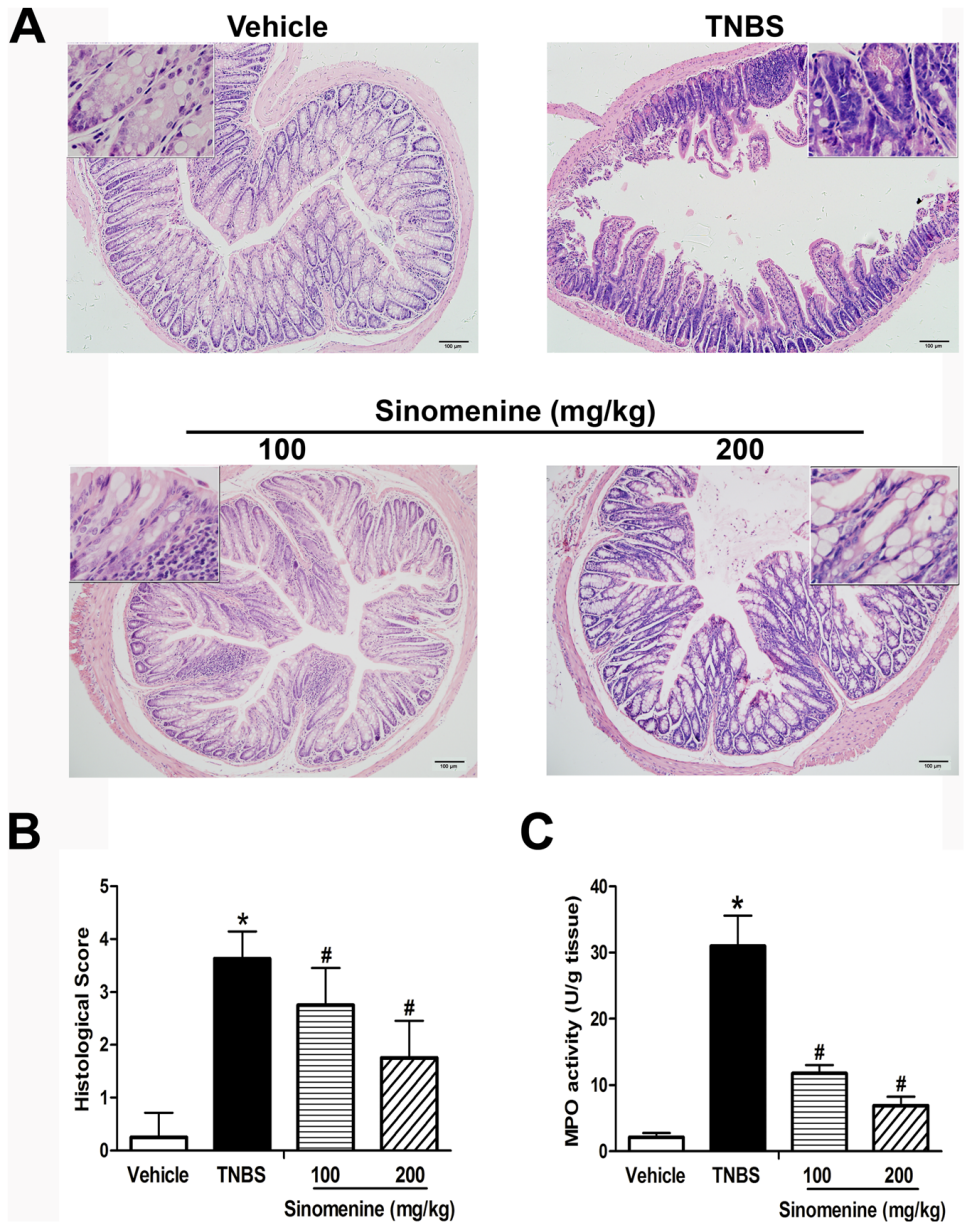
Effects on histological analysis and MPO activity measurement by sinomenine. Slices were inspected by a certified pathologist and the inflammatory severity of colonic tissues from survived mice in each group was scored and filmed. Samples from mice that were given sinomenine exhibited minor inflammatory reaction when compared with the TNBS group. **A.** Representative cross sections of the transversing colon. Magnifications of the images are 100-fold and 400-fold. Scale bar, 100 µm. **B.** Histological scores are depicted as means ± SEM. **C.** **Effect of sinomenine on MPO activity in TNBS-induced colitis.** Sinomenine significantly reduced the increased MPO activity in colon tissues induced by TNBS when compared with the vehicle. n = 6 per group, *p<0.05 versus the vehicle group and ^#^ *p*<0.05 versus the TNBS group.

The colons of the mice with TNBS-induced colitis showed large areas of ulceration, severe depletions of mucin-producing goblet and epithelial cells, thickening of the muscular layer, and high levels of leukocyte and polymorphonuclear (PMN) infiltration, as shown in Figure 2A. Administration of sinomenine (100 and 200 mg/kg) improved the above-mentioned signs and the histological scores, which decreased significantly by 24% (*p* = 0.015) and 52% (*p*<0.001), respectively (Figure 2B).

In the TNBS group, the MPO activity was significantly increased by 15-fold (*p*<0.001) compared with the vehicle group; the MPO activity in the mice treated with 100 mg/kg and 200 mg/kg sinomenine decreased by 63% (*p*<0.001) and 78% (*p*<0.001), respectively, compared with the TNBS group, as shown in Figure 2C. These results suggested that 200 mg/kg sinomenine played a more significant role in relieving histological symptoms, which presented as histological appearance, histological score and MPO activity.

### Sinomenine Down-regulates the Expression of miR-155, c-Maf, TNF-α and IFN-γ mRNAs

**Figure 3:**
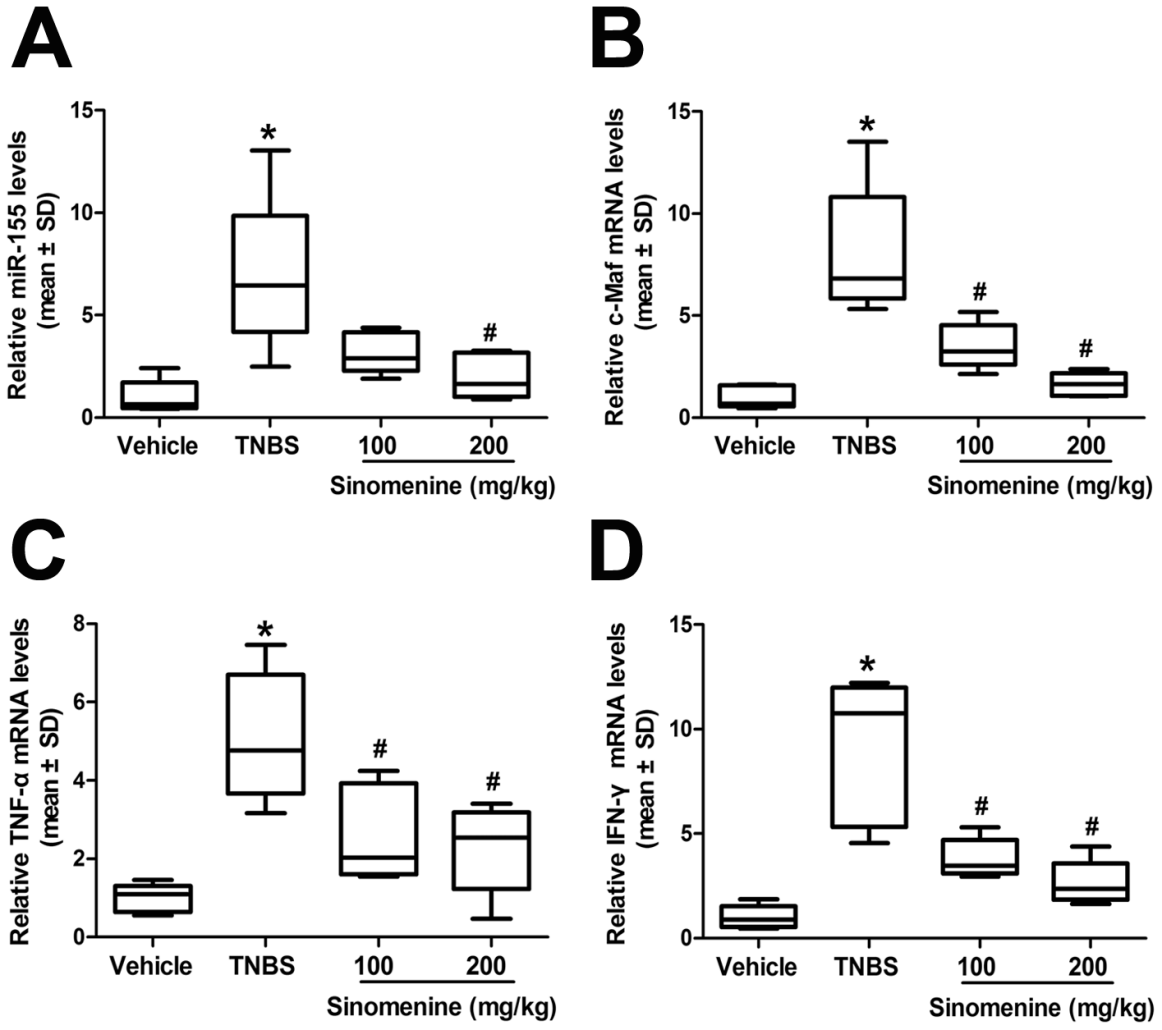
Reduction of miR-155, c-Maf, TNF-α and IFN-γ mRNA by sinomenine. Both dosages of sinomenine down-regulated the increased expression levels of miR-155 (**A**), c-Maf (**B**), TNF-α (**C**) and IFN-γ (**D**) which were induced by TNBS. n = 6 per group, *p<0.05 versus the vehicle group and ^#^ *p*<0.05 versus the TNBS group.

MiR-155 plays a crucial role in CD4^+^ T cell subset differentiation, and one of its known targets in CD4^+^ T cells is the transcription factor c-Maf [27]. Therefore, we assessed miR-155, c-Maf, TNF-α and IFN-γ mRNA expression levels using quantitative RT-PCR. In comparison with the vehicle, we observed that the expression levels of miR-155, c-Maf, TNF-α and IFN-γ were increased by 7-fold (*p* = 0.010), 8-fold (*p* = 0.001), 5-fold (*p* = 0.001) and 9-fold (*p* = 0.001), respectively, in the TNBS group. The expression of miR-155 was decreased by 54% (*p* = 0.067) in mice treated with 100 mg/kg sinomenine and was significantly decreased by 71% (*p* = 0.025) with 200 mg/kg sinomenine treatment compared with the TNBS group (Figure 3A). However, in comparison with the mice with TNBS-induced colitis, the mRNA levels of c-Maf, TNF-α and IFN-γ were significantly reduced by 56% (*p* = 0.018), 49% (*p* = 0.028) and 58% (*p* = 0.012), respectively, in mice administered 100 mg/kg sinomenine, and the levels were decreased by 80% (*p* = 0.002), 55% (*p* = 0.014) and 71% (*p* = 0.004), respectively, with 200 mg/kg sinomenine (Figures 3B, C, D). These results showed that sinomenine could down-regulate the expression of miR-155, c-Maf, TNF-α and IFN-γ mRNAs in a dose-dependent manner.

### Analysis of Expression Levels of c-Maf, TNF-α and IFN-γ Proteins by Immunohistochemistry

**Figure 4:**
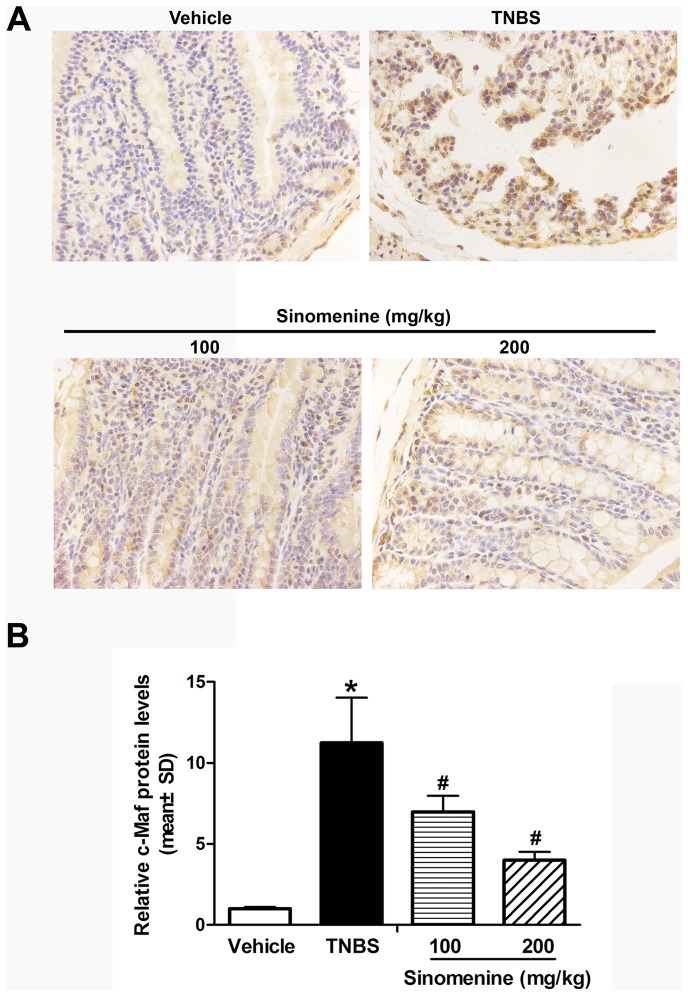
Effect of sinomenine on the protein levels of c-Maf. The protein levels of c-Maf increased in the TNBS group compared to the vehicle; however, c-Maf protein expression was significantly inhibited by sinomenine at doses of 100 and 200 mg/kg when compared with the TNBS-induced colitis in mice. n = 6 per group, *p<0.05 versus the vehicle group and ^#^ *p*<0.05 versus the TNBS group.

**Figure 5:**
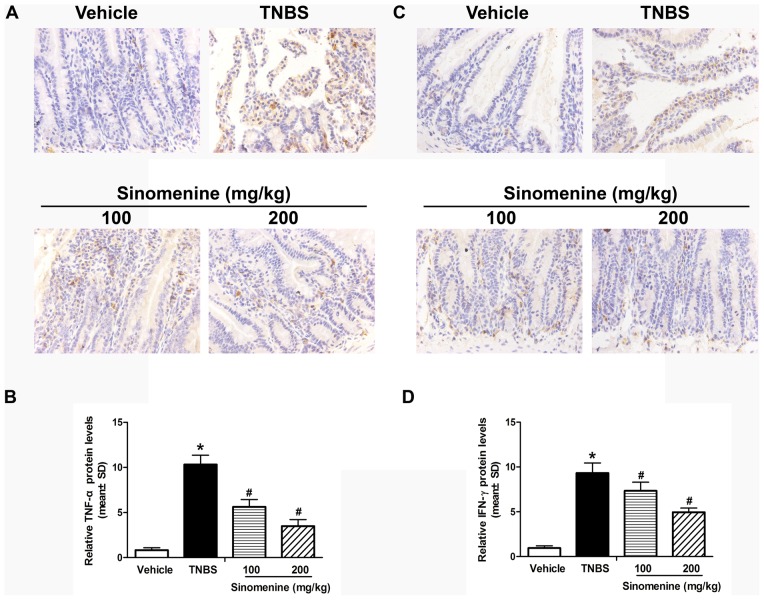
Effect on the protein levels of TNF-α and IFN-γ by sinomenine. The protein expression levels of TNF-α (A, B) and IFN-γ (C, D) increased in the TNBS group when compared to the vehicle group; nevertheless, they were reduced by sinomenine in doses of 100 and 200 mg/kg when compared with the TNBS groups. n = 6 per group, *p<0.05 versus the vehicle group and ^#^ *p*<0.05 versus the TNBS group.

To further study the regulatory effects of sinomenine on c-Maf, TNF-α and IFN-γ, we measured their protein levels using immunohistochemistry. As shown in Figure 4, the protein levels of c-Maf increased by 11-fold (*p*<0.001) in the TNBS group compared with the vehicle group; however, c-Maf protein expression was inhibited at 100 mg/kg sinomenine by 38% (*p* = 0.028) and at 200 mg/kg sinomenine by 64% (*p* = 0.002) compared with the TNBS group. We observed that the TNF-α and IFN-γ protein levels were higher by 12-fold (*p*<0.001) and 10-fold (*p*<0.001), respectively, in the TNBS group than in vehicle group. Nevertheless, the expression levels of these proteins were significantly decreased respectively by 45% (*p*<0.001) and 21% (*p* = 0.037) in 100 mg/kg sinomenine and significantly decreased respectively by 66% (*p*<0.001) and 47% (*p*<0.001) in 200 mg/kg sinomenine group when compared with the TNBS group (Figures 5).

### Correlation of miR-155 Expression with c-Maf, TNF-α and IFN-γ Levels

**Figure 6:**
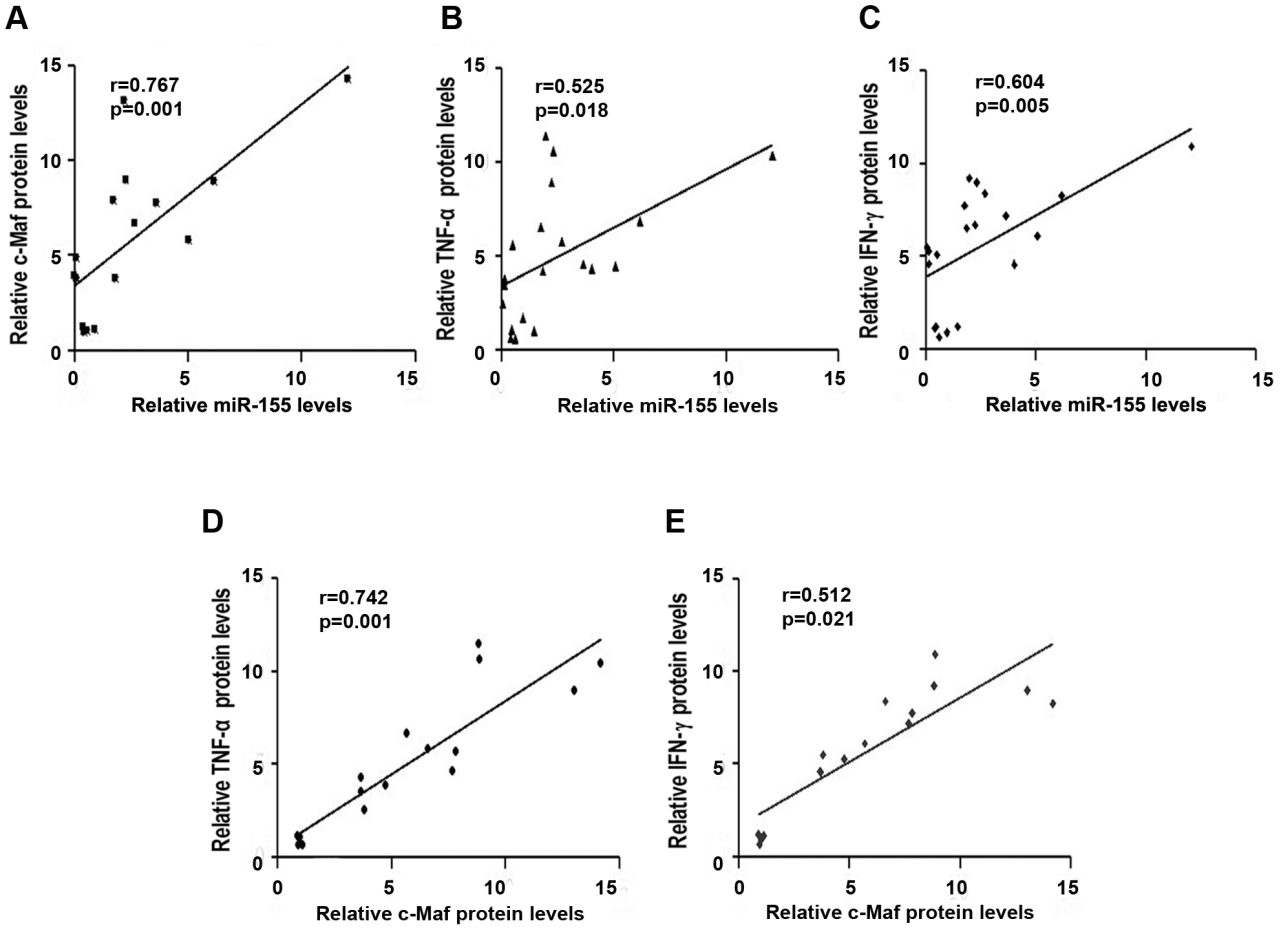
Correlation of miR-155 expression with c-Maf, TNF-α and IFN-γ. A, B, C. MiR-155 transcript expression had positive correlations with c-Maf protein, TNF-α protein, and IFN-γ protein expressions. **D, E.** The protein expression of c-Maf had positive correlations with TNF-α protein and IFN-γ protein expressions. Each data point represents one mouse in each panel, and each panel includes data from all mice in our experiments. Data were analyzed by Pearson’s correlation coefficient.

To gain insight into the mechanisms by which sinomenine down-regulated miR-155, c-Maf, TNF-α and IFN-γ expression, we investigated the correlation among the expression levels of miR-155, c-Maf, TNF-α and IFN-γ. The level of miR-155 transcript expression was significantly positively correlated with the levels of the c-Maf (r = 0.767, *p = *0.001), TNF-α (r = 0.525, *p = *0.018), and IFN-γ (r = 0.604, *p = *0.005) proteins (Figures 6A, B and C), but no correlations were found with the c-Maf (r = 0.342, *p = *0.140), TNF-α (r = 0.483, *p = *0.058), and IFN-γ (r = 0.474, *p = *0.064) mRNA levels (data not shown). As shown in Figures 6D and E, the level of c-Maf protein was significantly positively correlated with the levels of TNF-α protein (r = 0.742, *p = *0.001) and IFN-γ protein (r = 0.512, *p = *0.021) in all of the animals tested.

## Discussion

Although the exact etiology of IBD is still unclear, it is thought that altered immunological functions, because of the interplay between genetic susceptibility and certain environmental factors, can contribute to the mucosal inflammation of the intestinal tract [28]. The imbalances in the levels of pro-inflammatory cytokines have been implicated in the maintenance of the inflammatory response. As an animal model, rectal administration of TNBS is a well-characterized model of experimental colitis that is thought to resemble CD in disease presentation and cytokine profiles [7], [8].

In the present study, we used a murine model of TNBS-induced colitis to investigate the potential beneficial and protective effects of sinomenine on IBD and the underlying mechanisms involved. We found that sinomenine treatments at both 100 and 200 mg/kg improved TNBS-induced colitis symptoms, which presented as decreased body weights and survival rates and elevated diarrhea scores. At molecular and cellular levels, sinomenine decreased MPO activity and histological disease scores. Higher dosage of sinomenine (200 mg/kg) worked faster and was more efficacious at relieving these clinical symptoms. Importantly, we demonstrated for the first time that early administration of sinomenine could down-regulate the expression of miR-155 and c-Maf in mice with TNBS-induced colitis.

Sinomenine has been found to inhibit T-and B-lymphocyte activation, proliferation and function and to interfere with the function of several other cell types, such as dendritic cells (DC) [29]. Sinomenine inhibits the bone marrow-derived DC expression of Ia, CD86, and CD40; the production of IL-12, TNF-α, and IL-1β; and the antigen-presenting capacity in a dose-dependent manner [29]. The potential anti-inflammatory and immunosuppressive mechanisms of sinomenine have been investigated in multiple immune-related disorders in experimental animal models and in some clinical applications. It has been shown that sinomenine inhibits the proliferation of lymphocytes, suppress Th1 cell differentiation and reduces the secretion of prostaglandin E2 (PGE2), leukotriene C4 (LTC4), nitric oxide (NO) and tumor necrosis factor-α (TNF-α) from activated DC/macrophages and monocytes [30].

MiRNAs are thought to post-transcriptionally regulate gene expression by interacting with the RNA-induced silencing complex (RISC) and binding to complementary sequences in the 3′ untranslated regions (3′ UTRs) of target genes to prevent protein accumulation by repressing translation or by inducing mRNA degradation [31], [32]. Dysregulation of miRNAs has been observed in several autoimmune diseases [33], [34]. MiR-155 is processed from an exon of the non-coding RNA known as bic, its primary precursor [35]. Recent studies have shown that miR-155 over-expression leads to a bias in CD4^+^ T cells towards Th1 differentiation with IFN-γ and c-Maf involvement, although c-Maf has been identified as a direct target of miR-155 and as a T helper (Th) 2 cell-specific transcription factor that promotes the differentiation of Th2 cells [15], [27]. Furthermore, our data showed that miR-155 transcript expression was positively correlated with the protein levels but not mRNA levels of c-Maf and IFN-γ. Therefore, we hypothesized that sinomenine reduced the expression of miR-155 and c-Maf, and inhibited T-cell differentiation into Th1 cells, leading to a decrease in the production of IFN-γ, which ultimately alleviated the TNBS-induced colitis.

Considering that TNF-α plays an important role in the pathogenesis of CD and that miR-155 has been reported to affect the production of TNF-α at both the transcriptional and post-transcriptional levels [36], [37], we evaluated the mRNA and protein levels of TNF-α. Our results showed that both dosages (100 and 200 mg/kg) of sinomenine could suppress the expression of TNF-α to a remarkable degree in mice with TNBS-induced colitis. MiR-155 may directly target transcript coding for several proteins such as Fas-associated death domain protein (FADD), IκB kinase ε (IKKε), and the receptor (TNFR superfamily)-interacting serine-threonine kinase 1 (Ripk1), all of which are involved in activating LPS/TNF-α signaling and enhancing TNF-α translation [36]. MiR-155 may target the 3′-UTR of TNF transcripts to increase their stability and enhance translation at the post-transcriptional level [36]. The correlation between miR-155 expression and TNF-α level suggests that sinomenine-mediated effects on TNF-α may happen via miR-155 in TNBS-induced colitis.

We have demonstrated that sinomenine decreases the up-regulated expression of TNF-α and IFN-γ caused by TNBS, which is consistent with a previous study [22]. It is our hypothesis that these decreased expressions were consequences of sinomenine-mediated down-regulation of miR-155 and c-Maf. However, it is yet to be tested whether the decreases of miR-155 and other inflammatory factors in this study are direct consequential events of sinomenine treatment. MiR-155 knock-out/knock-down mice are needed in the future study to definitively define the exact function of miR-155 in sinomenine-mediated suppression of colitis.

In conclusion, this study has shown that sinomenine suppresses major pro-inflammatory cytokines TNF-α and IFN-γ, and attenuates TNBS-induced colitis probably through down-regulation of miR-155 and c-Maf. Our results suggest that the higher dose (200 mg/kg) of sinomenine may have a greater therapeutic value for IBD treatment.
